# Women in the HIV Care Continuum: Assessing Re-linking Women to Care and Maintaining Viral Suppression Postpartum in Philadelphia from 2012 to 2023

**DOI:** 10.1007/s10461-026-05096-4

**Published:** 2026-03-12

**Authors:** Kira J. Nightingale, Tanner Nassau, Antoneta Karaj, John H. Holmes, Yun Li, Florence M. Momplaisir, Kathleen A. Brady, Elizabeth D. Lowenthal

**Affiliations:** 1 Department of Biostatistics, Epidemiology, and Informatics, Perelman School of Medicine, University of Pennsylvania, Philadelphia, PA USA; 2 Division of HIV Health, Philadelphia Department of Public Health, Philadelphia, PA USA; 3 Department of Internal Medicine, Perelman School of Medicine, University of Pennsylvania, Philadelphia, PA USA; 4 Department of Pediatrics, Perelman School of Medicine, University of Pennsylvania, Philadelphia, PA USA; 5 Division of General Pediatrics and Global Health Center, The Children’s Hospital of Philadelphia, Philadelphia, PA USA

**Keywords:** HIV, Postpartum period, Women’s health, Case management, Continuity of patient care

## Abstract

The purpose of this study was to describe the HIV care continuum postpartum in a cohort of postpartum women with HIV (WWH) in Philadelphia, and assess the impact of the City’s perinatal case management (PCM) program on engagement in the HIV care continuum. A cohort of 937 mothers who gave birth between 1/1/2012 and 12/31/2023 and who lived, gave birth, or received perinatal and pediatric care in Philadelphia, PA were enrolled. Using public health surveillance data, the impact of PCM use on engagement in care at 90 days postpartum, retention in care at 1-year postpartum, and viral suppression at 1-year postpartum was assessed using logistic regression with robust standard errors. Overall, 51.1%, 65.4%, and 42.0% of the population was engaged in care at 90 days, and retained in care and virally suppressed at 1-year, respectively. A larger percentage of PCM users than non-users were engaged in care at 90 days (56.4% versus 43.9%) and retained in care (73.2% versus 59.2%) and virally suppressed (48.2% versus 37.1%) at 1 year. After controlling for confounders, PCM use was significantly associated with increased engagement in care at 90 days (aOR = 1.403 [1.030–1.913]), retention in care at 1 year (aOR = 1.576 [1.163–2.136]), and viral suppression at 1 year (aOR = 1.412 [1.060–1.881]). Philadelphia’s PCM program is effective in improving engagement in the HIV care continuum during the postpartum period. Other US cities, particularly those with high HIV prevalence, should consider adopting a similar city-funded PCM program for pregnant and postpartum WWH.

**Supplementary Information:**

The online version contains supplementary material available at https://doi.org/10.1007/s10461-026-05096-4.

## Introduction

The US has made significant progress in reducing mother-to-child transmission of HIV, with transmission rates dropping from 1.9 to 0.9 per 100,000 live births between 2010 and 2019 [1]. The focus on eliminating perinatal transmission resulted in heightened attention to HIV care during pregnancy, but studies that have examined postpartum HIV-related outcomes show a stark decline in care engagement between delivery and postpartum [2–5]. Postpartum engagement in HIV care is strongly associated with long-term retention in care, and poor retention in care is associated with viral rebound even among those who previously achieved viral suppression [3, 6]. Additional efforts are therefore needed to ensure continued engagement with HIV care during the postpartum period and maintain the long-term health of WWH during a uniquely vulnerable stage of life [7].

Barriers to HIV care are numerous, including lack of familial/societal support, health literacy, and HIV-related stigma particularly among healthcare workers [8, 9]. These barriers are compounded in postpartum HIV care, when additional factors such as depression and new time constraints become increasingly important [10, 11]. Case management programs can play a key role in overcoming challenges by linking patients with appropriate resources to meet their medical and personal needs, including housing, food, and transportation. However, not all programs are equally effective and the literature leaves considerable uncertainty regarding the best way to administer case management services [12, 13]. Further, effective care coordination between the pregnancy and postpartum periods can be difficult to achieve due to complexities in referral systems across institutions, the absence of process tools to help coordinate care, and lack of dedicated staff at healthcare facilities [14].

Philadelphia’s Perinatal Case Management (PCM) program, which began in 1992 and has been expanded and enhanced multiple times since, is a city health department-funded program that seeks to fill gaps in care coordination. It is an optional, referral-based program that connects pregnant and postpartum WWH with medical and social services based on a detailed assessment of participant needs at no cost to participants. Participants receive guidance and support from case managers who accompany WWH to appointments and make additional contact attempts for those at risk of falling out of care. Case managers also guide participants through obtaining public assistance, navigating housing issues, and securing referrals to legal services when needed. The PCM program seeks to reduce or eliminate barriers to care for WWH, recognizing that navigation of the medical system is complex and can often pose insurmountable challenges to patients experiencing additional barriers. Details of case manager qualifications and program administration have been described elsewhere [15]. The Philadelphia program is one of just a few city-funded case management programs in the United States, and has the potential to serve as a model for other cities across the country.

Engagement in prenatal care among pregnant WWH in Philadelphia has steadily increased over the past 10 years, with 92.5–98.6% of pregnant WWH receiving prenatal care in 2019–2023, compared to 78.4% in 2011–2015 [16, 17]. Receipt of antiretroviral therapy (ART) during pregnancy has similarly increased, from 67.5% in 2011–2015 to 86.5% in 2023 [16, 17]. Despite these advancements in HIV care during pregnancy, there is no recent data on postpartum HIV-related outcomes from Philadelphia, limiting our understanding of whether these improvements also apply to the postpartum period. A prior study conducted using HIV surveillance data from 2005 to 2013 found that Philadelphia’s PCM program was effective in increasing the likelihood of viral suppression at delivery and retention in care at 1 year postpartum [15]. Despite the program-related improvements, however, postpartum HIV care continuum outcomes overall were suboptimal, with only 39.9% of women reengaged in HIV care at 90 days postpartum, 43.9% retained in care at 1 year postpartum, and 35.7% virally suppressed at 1 year postpartum [15]. Given the significant improvements to HIV care since the last Philadelphia-based analysis, including advances in ART, the dissemination of the Undetectable = Untransmittable (U = U) campaign, and universal ART regardless of CD4 count, marked improvements in engagement in the postpartum HIV care continuum would be expected [18–20]. The PCM program has also been revised and expanded since the last analysis, with direct funding from the city to program administrators via a competitive proposal review process and updated program standards. In our study, we therefore aimed to address our gaps in knowledge regarding the current state of postpartum HIV care engagement by assessing engagement in care at 90 days, and retention in care and viral suppression at one year postpartum, and to elucidate the impact of Philadelphia’s updated PCM program through a comparison of outcomes among PCM users and non-users.

## Methods

### Study Population

This study included all WWH who experienced a live birth between January 1, 2012-December 31, 2023, and who lived, gave birth, or received perinatal and pediatric care services in Philadelphia, PA. Women who received an HIV diagnosis after 30 days postpartum, or who died or moved out of state within 90 days postpartum were excluded. Women who died or moved out of state within one year postpartum were excluded from the 1-year outcome assessments. For women with more than one live birth during the study period, each birth was included. Twins were included as a single mother-infant pair.

Data were obtained from Enhanced HIV/AIDS Reporting System (eHARS), a Centers for Disease Control and Prevention (CDC)-developed application that facilitates the transmission of de-identified HIV surveillance data from local public health departments to the CDC. eHARS incorporates data from laboratory testing, pediatric HIV surveillance, vital statistics, and hospital medical records [21]. Information on PCM participation was obtained using Ryan White CAREWare software, an electronic health and social support services information system that was developed by the Health Resources and Services Administration (HRSA) and is used by the city of Philadelphia to monitor the use of services for people with HIV. This study was approved by the Philadelphia Department of Public Health Institutional Review Board (FWA00003616) with a waiver of informed consent.

### Outcome Measures

Three binary outcomes were assessed: (1) Engagement in care 90 days postpartum (90-day outcome); (2) Retention in care one year postpartum (1-year outcome); and (3) Viral suppression one year postpartum (1-year outcome). Using laboratory testing as a proxy for engagement in care, engagement in care at 90 days postpartum was defined as having at least one HIV viral load (VL) or CD4 test performed between one week and 90 days post-delivery [22]. Retention in care at one year was defined as having ≥ two VL/CD4 tests performed in the 12 months after delivery with ≥ 90 days between tests [23]. Viral suppression was defined as having a viral load ≤ 200 copies/mL at one year postpartum ± 60 days. When multiple viral load measurements were available within this timeframe, the result closest to one year was selected. Women without VL data during this period were classified in the primary analysis as having detectable VLs. A secondary analysis used a carry-forward approach in which the most recent VL result after 90 days postpartum was carried forward for women missing 1-year data, mirroring CDC reporting metrics which use the most recent HIV viral load result in a calendar year to determine suppression [24].

### Exposure

PCM use was the exposure of interest. For the 90-day outcome, mothers were classified as PCM users if they had at least two documented encounters with a case manager during pregnancy through 90 days postpartum. For the 1-year outcomes, use of PCM services was defined as having at least two documented encounters with a case manager from 90 days to one year postpartum. There is no standardized definition in the literature of what constitutes meaningful PCM use; we therefore based the requirement of two or more encounters on the prior analysis of Philadelphia’s PCM program [15]. Sensitivity analyses varied the number of required encounters in the specified period. For 1-year outcomes, an additional sensitivity analysis defined PCM use as at least two encounters between postpartum days 270–365, under the hypothesis that the closer PCM use occurs in relation to the outcome, the more likely it is to have an impact.

### Covariates

Potential covariates were selected based on existing literature and their suspected relationship with the exposure and/or outcomes [25]. These included maternal age at delivery (16–24, 25–34, or ≥ 35), race and ethnicity, country of birth (US/non-US), HIV diagnosis during vs. prior to pregnancy, single/multiple birth, parity, prior pregnancy while living with HIV, substance use during pregnancy (illicit drugs, alcohol, or tobacco), maternal HIV risk factor (heterosexual contact, injection drug use, perinatal exposure, or other), and Social Vulnerability Index (SVI) based on the mother’s last known address prior to delivery. Receipt of ART during pregnancy, maternal AIDS status, infant serostatus, ART provided to infant, and adequacy of prenatal care using both the Kessner and the Adequacy of Prenatal Care Utilization indices (APNCU) were described but not considered for inclusion in adjusted models due to their occurrence after PCM exposure [26]. Viral suppression at delivery was considered for inclusion in the 1-year outcomes, but not the 90-day outcome for the same reason. The Kessner index was included for historical comparison, and the APNCU as the currently preferred metric.

The SVI is a composite measure that has been used widely as a metric in health research to account for socioeconomic status [27]. Recognizing the immense impact of the COVID-19 pandemic on the delivery and receipt of HIV-related healthcare, a sensitivity analysis included a COVID-period indicator variable in the final adjusted models [28].

### Analysis

Demographic characteristics were compared using χ^2^ and t-tests, with Bonferroni post hoc tests where appropriate. Unadjusted outcomes were assessed with χ^2^ tests. Logistic regressions were used to assess adjusted outcomes. To account for repeated births from some mothers, robust sandwich estimators with clustering at the level of the mother were used to calculate standard errors [29]. Initial models were run using all eligible covariates of interest. Variables with > 15% of participants classified as “unknown” were not considered for inclusion in adjusted models. Backwards selection via the MASS package in R was used for model refinement, while retaining variables suspected to be confounders based on a priori knowledge [30]. All data cleaning and analysis was performed using R version 4.4.1.

## Results

A total of 962 infants and 691 mothers were eligible for inclusion (see Supplemental Material 1 for the consort diagram of excluded individuals). This included 50 twins, only one of whom from each pair was included in the cohort, resulting in a total of 937 mother-infant pairs for 90-day retention in care, 543 (58%) of whom used PCM services through 90 days postpartum. There were 11 mother-infant pairs who moved out of state and four maternal deaths within a year postpartum, resulting in 922 included for 1-year outcomes. Between 90 days and one year postpartum, 407 (44.1%) women used PCM services.

The study cohort was predominantly Black/African American (74.6%), aged 25–34 (60.2%), and resided in Philadelphia at the time of delivery (74.0%). Individuals who used PCM services during pregnancy through 90 days postpartum were significantly more likely to be under 35 years of age, Black/African American versus white, residents of Philadelphia, have had a previous birth while living with HIV, have more prior live births, have used ART during pregnancy, and have a higher SVI than those who didn’t use PCM services. Demographic differences in PCM use through one year postpartum were similar, although differences in maternal age at delivery and prior births while living with HIV were no longer statistically significant. Complete demographic characteristics can be found in Tables 1a and b.

**Table 1:** Sociodemographic characteristics of women living with HIV who lived, gave birth, or received prenatal/pediatric care in Philadelphia, PA between January 1, 2012–December 31, 2023 who were included in the analysis of engagement in care at 90 days (Table 1a) and 1-year outcomes (Table 1b), by PCM use

(a)	No PCM use	PCM use	Overall	P-value
	N = 394	N = 543	N = 937	
Maternal age at delivery				** < 0.001**
16–24	58 (14.7%)	89 (16.4%)	147 (15.7%)	
25–34	214 (54.3%)	350 (64.5%)	564 (60.2%)	
35+	122 (31.0%)	104 (19.2%)	226 (24.1%)	
*Maternal race and ethnicity*				** < 0.001**
Black/African American	268 (68.0%)	431 (79.4%)	699 (74.6%)	
Hispanic/Latino, all races	44 (11.2%)	47 (8.7%)	91 (9.7%)	
White	59 (15.0%)	41 (7.6%)	100 (10.7%)	
Other	23 (5.8%)	24 (4.4%)	47 (5.0%)	
*Maternal country of birth*				0.072
US-born	265 (67.3%)	385 (70.9%)	650 (69.4%)	
Non-US born	73 (18.5%)	107 (19.7%)	180 (19.2%)	
Unknown	56 (14.2%)	51 (9.4%)	107 (11.4%)	
*Single or multiple birth*				1
Single	383 (97.2%)	529 (97.4%)	912 (97.3%)	
Twin	11 (2.8%)	14 (2.6%)	25 (2.7%)	
*Resident of Philadelphia*				** < 0.001**
Yes	251 (63.7%)	442 (81.4%)	693 (74.0%)	
No	143 (36.3%)	101 (18.6%)	244 (26.0%)	
*Social Vulnerability Index*				** < 0.001**
	0.712 (0.261)	0.788 (0.204)	0.756 (0.232)	
Missing	*	*	10 (1.1%)	
*Previous birth(s) with HIV diagnosis*				** < 0.001**
First live birth w/ HIV	277 (70.3%)	321 (59.1%)	598 (63.8%)	
Previous live birth w/ HIV	104 (26.4%)	209 (38.5%)	313 (33.4%)	
Unknown	13 (3.3%)	13 (2.4%)	26 (2.8%)	
*Number of previous live births*				**0.002**
Mean (SD)	1.85 (1.38)	2.13 (1.40)	2.01 (1.40)	
Missing	11 (2.8%)	10 (1.9%)	26 (2.8%)	
*Maternal HIV risk factor*				0.153
Heterosexual contact	312 (79.2%)	427 (78.6%)	739 (78.9%)	
IV drug use with/without heterosexual contact	49 (12.4%)	57 (10.5%)	106 (11.3%)	
Perinatal exposure	25 (6.3%)	53 (9.8%)	78 (8.3%)	
Other	*	*	14 (1.5%)	
*Timing of HIV diagnosis*				0.321
Before pregnancy	343 (87.1%)	459 (84.5%)	802 (85.6%)	
During current pregnancy	51 (12.9%)	84 (15.5%)	135 (14.4%)	
*Detectable viral load at delivery*				0.15
Yes	67 (17.0%)	73 (13.4%)	140 (14.9%)	
No	325 (82.5%)	469 (86.4%)	794 (84.7%)	
Missing	*	*	*	
*Tobacco use during pregnancy*				**0.003**
Yes	24 (6.1%)	43 (7.9%)	67 (7.2%)	
No	110 (27.9%)	201 (37.0%)	311 (33.2%)	
Unknown	260 (66.0%)	299 (55.1%)	559 (59.7%)	
*Alcohol use during pregnancy*				**0.003**
Yes	*	*	23 (2.5%)	
No	126 (32.0%)	229 (42.2%)	355 (37.9%)	
Unknown	260 (66.0%)	299 (55.1%)	559 (59.7%)	
*Other substance use during pregnancy*				**0.003**
Yes	42 (10.7%)	67 (12.3%)	109 (11.6%)	
No	92 (23.4%)	177 (32.6%)	269 (28.7%)	
Unknown	260 (66.0%)	299 (55.1%)	559 (59.7%)	
*Use of ART during pregnancy*				**0.001**
Yes	335 (85.0%)	500 (92.1%)	835 (89.1%)	
No	15 (3.8%)	6 (1.1%)	21 (2.2%)	
Unknown	44 (11.2%)	37 (6.8%)	81 (8.6%)	
*AIDS diagnosis*				0.478
AIDS diagnosed before pregnancy	96 (24.4%)	128 (23.6%)	224 (23.9%)	
AIDS diagnosed during pregnancy	12 (3.0%)	22 (4.1%)	34 (3.6%)	
AIDS diagnosed within 1 year postpartum	*	*	*	
No AIDS diagnosis	283 (71.8%)	392 (72.2%)	675 (72.0%)	
*Infant serostatus*				0.106
Definitively negative	360 (91.4%)	514 (94.7%)	874 (93.3%)	
Pediatric HIV	1 (0.3%)	2 (0.4%)	3 (0.3%)	
Perinatal HIV exposure, final status unknown	33 (8.3%)	27 (4.9%)	60 (6.4%)	
*Kessner Index*				0.296
Adequate	133 (33.8%)	204 (37.6%)	337 (36.0%)	
Intermediate	81 (20.6%)	123 (22.7%)	204 (21.8%)	
Inadequate	32 (8.1%)	44 (8.1%)	76 (8.1%)	
Missing	148 (37.6%)	172 (31.7%)	320 (34.2%)	
*APNCU*				0.294
Adequate plus	12 (3.0%)	15 (2.8%)	27 (2.9%)	
Adequate	60 (15.2%)	91 (16.8%)	151 (16.1%)	
Intermediate	57 (14.5%)	97 (17.9%)	154 (16.4%)	
Inadequate	50 (12.7%)	81 (14.9%)	131 (14.0%)	
Missing	215 (54.6%)	259 (47.7%)	474 (50.6%)	
*ART provided to infant*				0.723
Yes	383 (97.2%)	531 (97.8%)	914 (97.5%)	
Unknown	11 (2.8%)	12 (2.2%)	23 (2.5%)	

(b)	No PCM use	PCM use	Overall	P-value
	N = 515	N = 407	N = 922	
*Maternal age at delivery*				0.066
16–24	76 (14.8%)	70 (17.2%)	146 (15.8%)	
25–34	302 (58.6%)	255 (62.7%)	557 (60.4%)	
35+	137 (26.6%)	82 (20.1%)	219 (23.8%)	
*Maternal race and ethnicity*				** < 0.001**
Black/African American	357 (69.3%)	332 (81.6%)	689 (74.7%)	
Hispanic/Latino, All races	50 (9.7%)	39 (9.6%)	89 (9.7%)	
White	78 (15.1%)	21 (5.2%)	99 (10.7%)	
Other	30 (5.8%)	15 (3.7%)	45 (4.9%)	
*Maternal country of birth*				**0.046**
US-born	356 (69.1%)	284 (69.8%)	640 (69.4%)	
Non-US born	91 (17.7%)	88 (21.6%)	179 (19.4%)	
Unknown	68 (13.2%)	35 (8.6%)	103 (11.2%)	
Single or multiple birth				0.85
Single	502 (97.5%)	395 (97.1%)	897 (97.3%)	
Twin	13 (2.5%)	12 (2.9%)	25 (2.7%)	
*Resident of Philadelphia*				** < 0.001**
Yes	352 (68.3%)	335 (82.3%)	687 (74.5%)	
No	163 (31.7%)	72 (17.7%)	235 (25.5%)	
*Social Vulnerability Index*				** < 0.001**
	0.726 (0.254)	0.797 (0.190)	0.758 (0.231)	
Missing	*	*	10 (1.1%)	
*Previous birth(s) with HIV diagnosis*				**0.047**
First live birth w/ HIV	342 (66.4%)	246 (60.4%)	588 (63.8%)	
Previous live birth w/ HIV	157 (30.5%)	151 (37.1%)	308 (33.4%)	
Unknown	16 (3.1%)	10 (2.5%)	26 (2.8%)	
*Number of previous live births*				**0.001**
Mean (SD)	1.88 (1.36)	2.18 (1.43)	2.01 (1.40)	
Missing	*	*	21 (2.3%)	
*Maternal HIV risk factor*				0.282
Heterosexual contact	398 (77.3%)	329 (80.8%)	727 (78.9%)	
IV drug use with/without heterosexual contact	68 (13.2%)	37 (9.1%)	105 (11.4%)	
Perinatal exposure	42 (8.2%)	35 (8.6%)	77 (8.4%)	
Other	*	*	13 (1.4%)	
*Timing of HIV diagnosis*				0.269
Before pregnancy	449 (87.2%)	340 (83.5%)	789 (85.6%)	
During current pregnancy	66 (12.8%)	67 (16.5%)	133 (14.4%)	
*Detectable viral load at delivery*				0.115
Yes	87 (16.7%)	52 (12.8%)	138 (15.0%)	
No	428 (83.1%)	355 (87.2%)	783 (84.9%)	
Missing	*	*	*	
*Tobacco use during pregnancy*				**0.013**
Yes	38 (7.4%)	27 (6.6%)	65 (7.0%)	
No	150 (29.1%)	156 (38.3%)	306 (33.2%)	
Unknown	327 (63.5%)	224 (55.0%)	551 (59.8%)	
*Alcohol use during pregnancy*				**0.015**
Yes	*	*	22 (2.4%)	
No	174 (33.8%)	175 (43.0%)	349 (37.9%)	
Unknown	327 (63.5%)	224 (55.0%)	551 (59.8%)	
*Other substance use during pregnancy*				** < 0.001**
Yes	68 (13.2%)	37 (9.1%)	105 (11.4%)	
No	120 (23.3%)	146 (35.9%)	266 (28.9%)	
Unknown	327 (63.5%)	224 (55.0%)	551 (59.8%)	
*Use of ART during pregnancy*				**0.003**
Yes	444 (86.2%)	377 (92.6%)	821 (89.0%)	
No	17 (3.3%)	3 (0.7%)	20 (2.2%)	
Unknown	54 (10.5%)	27 (6.6%)	81 (8.8%)	
AIDS diagnosis				0.781
AIDS diagnosed before pregnancy	125 (24.3%)	93 (22.9%)	218 (23.6%)	
AIDS diagnosed during pregnancy	17 (3.3%)	16 (3.9%)	33 (3.6%)	
AIDS diagnosed within 1 year postpartum	*	*	*	
No AIDS diagnosis	370 (71.8%)	297 (73.0%)	667 (72.3%)	
*Infant Serostatus*				0.132
Definitively negative	474 (92.0%)	388 (95.4%)	862 (93.5%)	
Pediatric HIV	2 (0.4%)	1 (0.2%)	3 (0.3%)	
Perinatal HIV exposure, final status unknown	39 (7.6%)	18 (4.4%)	57 (6.2%)	
*Kessner Index*				0.1
Adequate	174 (33.8%)	157 (38.6%)	331 (35.9%)	
Intermediate	106 (20.6%)	97 (23.8%)	203 (22.0%)	
Inadequate	46 (8.9%)	27 (6.6%)	73 (7.9%)	
Missing	189 (36.7%)	126 (31.0%)	315 (34.2%)	
*APNCU*				0.196
Adequate plus	15 (2.9%)	12 (2.9%)	27 (2.9%)	
Adequate	76 (14.8%)	73 (17.9%)	149 (16.2%)	
Intermediate	77 (15.05%)	73 (17.9%)	150 (16.3%)	
Inadequate	68 (13.2%)	61 (15.0%)	129 (14.0%)	
Missing	279 (54.2%)	188 (46.2%)	467 (50.7%)	
*ART provided to infant*				0.927
Yes	502 (97.5%)	398 (97.8%)	900 (97.6%)	
Unknown	13 (2.5%)	9 (2.2%)	22 (2.4%)	

In compliance with the Department of Public Health policy, all cell counts < 10 have been suppressed, other than those already available through public health reports. Suppressed cells are indicated with an asterisk (*). Overall percentages may not sum to 100% due to cell suppression. PCM use was defined as at least two documented encounters during pregnancy and through 90 days postpartum for the 90-day outcome, and as at least two documented encounters from 90 days through 1-year postpartum for the 1-year outcomes. Women were censored for the 1-year outcomes if they died or moved out of Pennsylvania within 1-year postpartum. “Maternal HIV risk factor – Other” includes the CDC risk factor classifications of “other exposures,” “no identified risk factor,” and “no risk factor reported.” The Kessner Index uses the following definitions: (1) adequate prenatal care—at least nine prenatal care visits during a full term pregnancy with the initial prenatal care visit during the first trimester, (2) inadequate prenatal care—fewer than five prenatal care visits occurred after 18 weeks of gestation or the initial prenatal care visit took place during the third trimester, and (3) intermediate prenatal care—any other combination of number of visits and timing of first visit [17]. The APNCU classifies prenatal care based on time of initiation (adequacy of initiation) and the number of prenatal visits attended (adequacy of received services). Adequacy of initiation is classified into four groups: pregnancy months 1–2, pregnancy months 3–4, pregnancy months 5–6, and pregnancy months 7–9. Adequacy of received services is based on the recommended number of prenatal care visits for an uncomplicated pregnancy in the American College of Obstetricians and Gynecologists standards, and is classified as follows: Inadequate (less than 50% of recommended visits), Intermediate (50–70% of recommended visits), Adequate (80–109% of recommended visits), and Adequate Plus (110% or more of recommended visits). These two factors are then combined into the final APNCU score: (1) adequate plus—prenatal care began by the 4th month and adequacy of received services is adequate plus; (2) adequate—prenatal care began by the 4th month and adequacy of received services is adequate; (3) intermediate—prenatal care began by the 4th month and adequacy of received services is intermediate; and (4) inadequate—prenatal care began after the 4th month or adequacy of received services is inadequate [17]. Differences in characteristics between PCM users and non-users were assessed using χ^2^ and t-tests, with Bonferroni post hoc tests as appropriate. A threshold of p ≤ 0.05 was used for statistical significance (bolded). The following variables were not considered for inclusion in adjusted models because they occurred after PCM exposure: viral load at delivery, use of ART during pregnancy, infant serostatus, Kessner index, APNCU index, and ART provided to infant. Tobacco, alcohol, and other substance use was not considered for inclusion in the model due to a high number of participants classified as “unknown.”

### Engagement in Care at 90-Days Postpartum

**Fig. 1:**
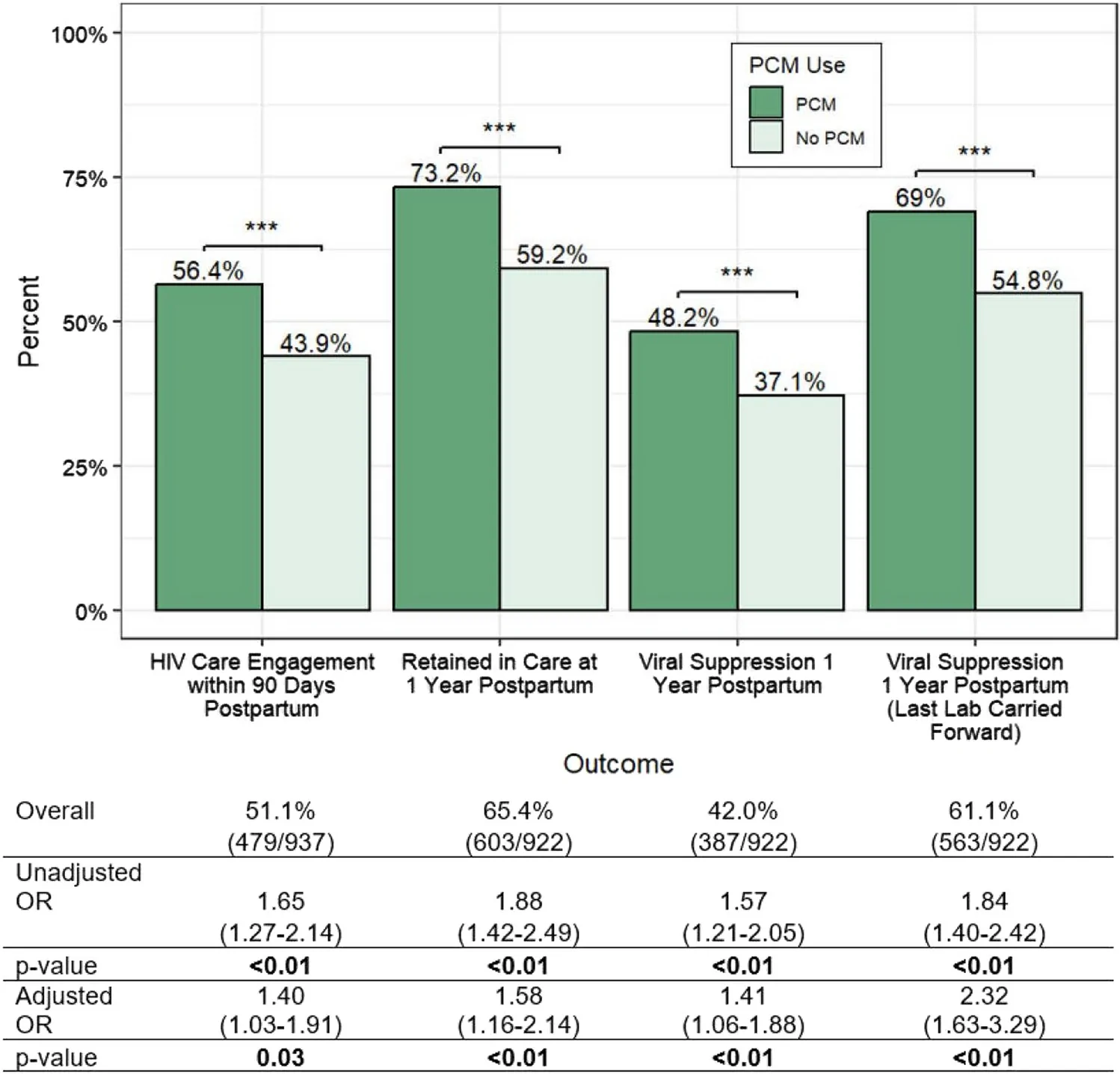
HIV care continuum outcomes of women with HIV who lived, gave birth, or received prenatal/pediatric care in Philadelphia, PA between January 1, 2012-December 31, 2023, by PCM use. The bar graph shows percentages of women achieving each outcome, with the overall percentage, unadjusted odds ratio, and p-value for the group-wise comparison listed below. All outcomes, both adjusted and unadjusted, were statistically significantly different between groups, with PCM users having better outcomes

Overall, 51.1% of individuals engaged in HIV care within 90-days postpartum. In the unadjusted analysis, individuals who used PCM were significantly more likely to be engaged in care at 90 days compared to those who did not use PCM (56.4% versus 43.9%, p < 0.01, Fig. 1). After adjusting for race, maternal birth country, Philadelphia residency, maternal HIV risk factor, and SVI, individuals who used PCM services were significantly more likely than those who did not use PCM services to be engaged in care at 90 days postpartum (aOR = 1.403 [1.030–1.913], p = 0.03, Table 2). An interaction term between PCM use and SVI was assessed but was not statistically significant (p = 0.09) and its inclusion in the model had no impact on the effect estimate of PCM use; it was therefore not included in the final model.

**Table 2:** Adjusted analysis of the effect of PCM use on engagement in care 90 days postpartum in a cohort of women living with HIV who lived, gave birth, or received prenatal/pediatric care in Philadelphia, PA between January 1, 2012–December 31, 2023

	aOR	95% CI	P-value
*PCM use*			
No	Ref		
Yes	1.403	1.030–1.913	**0.032**
*Race*			
White	Ref		
Black/African American	1.549	0.849–2.826	0.154
Hispanic/Latino	1.347	0.630–2.876	0.442
Other	2.567	0.974–6.763	0.056
*Mother's birth country*			
US Born	Ref		
Non-US Born	1.501	0.947–2.380	0.084
Unknown	0.785	0.471–1.310	0.354
*Philadelphia resident*			
No	Ref		
Yes	1.82	1.190–2.782	**0.006**
*Social Vulnerability Index*	0.817	0.394–1.697	0.588
*Maternal HIV risk*			
Heterosexual contact	Ref		
IV drug use with/without heterosexual contact	1.367	0.769–2.432	0.287
Perinatal exposure	3.414	1.766–6.597	** < 0.001**
Other	1.34	0.363–4.944	0.66

The effect of PCM use was assessed using logistic regression with robust standard errors to account for multiple births from the same woman during the study period. A threshold of p ≤ 0.05 was used for statistical significance (bolded). Additional variables that were assessed for but not included in the final model were maternal age at delivery, HIV diagnosis during vs. prior to pregnancy, single/multiple birth, parity, and prior pregnancy while living with HIV.

### Retention in Care at 1 Year Postpartum

Retention in care at 1 year postpartum was higher than retention at 90 days, with 65.4% of individuals meeting the one-year definition. Significantly more individuals who used PCM services (73.2%) were retained in care at 1 year as compared to those who did not use PCM services (59.2%, p < 0.01, Fig. 1). PCM users were significantly more likely to be retained in care at 1 year than non-users after adjusting for race, maternal birth country, Philadelphia residency, SVI, prior pregnancy with HIV, maternal HIV risk factor, and viral load at delivery (aOR = 1.576 [1.163–2.136], p < 0.01, Table 3).

**Table 3:** Adjusted analyses of the effect of PCM use on retention in care and viral suppression at 1 year postpartum in a cohort of women living with HIV who lived, gave birth, or received prenatal/pediatric care in Philadelphia, PA between January 1, 2012–December 31, 2023

	Retention in care 1 year postpartum			Viral suppression at 1 year postpartum (a)			Viral suppression at 1 year − carry forward last lab (b)		
	aOR	95% CI	P-value	aOR	95% CI	P-value	aOR	95% CI	P-value
*PCM use*									
No	Ref			Ref			Ref		
Yes	1.576	1.163–2.136	**0.003**	1.412	1.060–1.881	**0.018**	1.752	1.277–2.404	** < 0.001**
*Race*									
White	Ref			Ref			Ref		
Black/African American	1.331	0.787–2.251	0.286	0.843	0.484–1.468	0.547	0.673	0.395–1.145	0.144
Hispanic/Latino	1.284	0.644–2.560	0.478	0.816	0.404–1.647	0.57	0.502	0.258–0.978	**0.043**
Other	1.03	0.471–2.250	0.942	0.955	0.437–2.087	0.908	0.773	0.330–1.812	0.553
*Mother's birth country*									
US born	Ref			Ref			Ref		
Non-US born	1.642	1.067–2.526	**0.024**	0.987	0.675–1.443	0.946	1.213	0.783–1.881	0.387
Unknown	1.236	0.723–2.111	0.439	1.237	0.770–1.989	0.379	1.057	0.637–1.755	0.829
*Philadelphia resident*									
No	Ref			Ref			Ref		
Yes	2.21	1.482–3.296	** < 0.001**	1.89	1.259–2.838	**0.002**	1.33	0.883–2.004	0.172
*Social Vulnerability Index*	0.784	0.378–1.625	0.513	0.793	0.392–1.606	0.519	1.025	0.503–2.092	0.945
*Prior pregnancy w/ HIV*									
No	Ref			Ref			Ref		
Yes	0.93	0.685–1.264	0.644	1.252	0.932–1.682	0.136	1.425	1.028–1.977	**0.034**
*Maternal HIV risk*									
Heterosexual contact	Ref			Ref			Ref		
IV drug use with/without heterosexual contact	1.097	0.671–1.793	0.712	0.337	0.183–0.619	** < 0.001**	0.552	0.333–0.914	**0.021**
Perinatal exposure	2.941	1.417–6.103	**0.004**	1.04	0.546–1.981	0.904	0.942	0.521–1.705	0.844
Other	1.39	0.540–3.576	0.494	0.755	0.221–2.581	0.654	0.463	0.152–1.411	0.176
Detectable VL at delivery									
No	Ref			Ref			Ref		
Yes	0.538	0.361–0.804	**0.002**	0.168	0.095–0.295	** < 0.001**	0.117	0.072–0.189	** < 0.001**

Viral suppression model (a) used viral load results at 1 year ± 2 months postpartum, with individuals without a lab result classified as unsuppressed. Model (b) used the last available lab after 90 days postpartum if there was not a viral load result at 1 year ± 2 months postpartum, with individuals without a lab result after 90 days postpartum classified as unsuppressed. The effect of PCM use on the outcomes was assessed using logistic regression with robust standard errors to account for multiple births from the same woman during the study period. A threshold of p ≤ 0.05 was used for statistical significance (bolded). Additional variables that were assessed for but not included in the final model were maternal age at delivery, HIV diagnosis during vs. prior to pregnancy, single/multiple birth, and parity.

### Viral Suppression at 1 Year Postpartum

Less than half of individuals (42.0%) were virally suppressed at 1 year postpartum. Among only those who had labs available for review at 1 year ± 2 months (495/922, 53.7%), 78.2% achieved viral suppression. Among all individuals, those who used PCM services were significantly more likely to have an undetectable viral load than those who did not use PCM services (48.2% versus 37.1%, p < 0.01, Fig. 1). This association remained after adjusting for race, maternal birth country, Philadelphia residency, SVI, prior pregnancy with HIV, maternal HIV risk factor, and viral load at delivery (aOR = 1.412 [1.060–1.881], p = 0.03, Table 3). Carrying forward the last available laboratory value after 90 days postpartum for women without a viral load result at one year resulted in a total of 609 (66.1%) women achieving viral suppression, with a significant association between PCM use and viral suppression in the unadjusted (69.0% versus 54.8%, p < 0.01, Fig. 1) and adjusted (aOR = 2.315 [1.631–3.285], p < 0.01, Table 3) analyses.

### Sensitivity Analyses

Sensitivity analyses adjusting the number of PCM encounters required to meet the definition of PCM use produced results generally consistent with the primary analysis (Supplemental Material 2). Categorizing PCM use as two or more encounters during days 270–365 produced similar results, with higher effect estimates (Supplemental Material 3). Results of the adjusted analyses including an indicator variable for the COVID period showed that COVID-19 was significantly associated with a reduction in the likelihood of women being retained in care at 90-days and 1-year, but not with viral suppression at one year. Regardless of COVID, PCM use remained significantly associated with all three outcomes (Supplemental Material 4).

## Discussion

Results from this study show that Philadelphia’s PCM program is associated with increased retention in care and viral suppression during the postpartum period. This unique, city-funded program represents an innovative and effective means of improving the health and well-being of WWH through the provision of supportive services during a time when women are uniquely vulnerable.

The factors associated with suboptimal engagement in HIV care postpartum are multifactorial, with studies finding significant associations between HIV care and depression, mental bandwidth, substance use, prior engagement in prenatal and HIV care, viral load during pregnancy, and timing of HIV diagnosis and initiation of ART [31–34]. Comprehensive case management programs are therefore an ideal intervention to support women during this vulnerable time, as they not only facilitate medical care but can link participants to social support services. However, case management programs require significant time and financial resources, and not all programs result in improved outcomes [35]. A study of HIV prevention case management programs in San Francisco found that a lack of standardized protocols and inadequate staff training were barriers to the provision of effective case management, but that the implementation of program standards and increased collaboration with public health authorities improved program performance [36]. A systematic review by Joo and Huber identified five key barriers to successful case management administration: including diverse and complex case management activities, insufficient training, poor collaboration with other health-care providers, and client relationship challenges [35]. A city-funded model such as Philadelphia’s provides the opportunity to overcome some of these barriers through consistent training on standardized protocols while housing case managers outside of a single healthcare system to increase inter-institution care coordination. Further, the provision of city funding limits strain on individual institutions that might be unable to provide similar no-cost services cost to patients.

Postpartum care continuum outcomes have improved since the last analysis using data from births occurring between 2005 and 2013 [15]. Despite the improvements over time and the success of this PCM program in improving retention in care and viral suppression, overall rates of all outcomes were suboptimal. However, retention in care in our population was higher than the national rate for all WWH (53.4%) as of the most recently available data [37]. Using a similar metric to the one used by the CDC, the percentage of women in our cohort achieving viral suppression was similar to that of all WWH in the US (63.5%) [37]. Few prior studies have assessed postpartum outcomes among WWH in the US. The most recent of these included births through the mid-2010s, and reported retention in care and viral suppression at one year between 43–70% and 38–41%, respectively [2, 38, 39]. The results of this study are similar, indicating that despite the advent of more effective ART and public health initiatives, there is still significant progress to be made in improving postpartum outcomes among WWH [19]. While some studies have assessed interventions to improve HIV care engagement during the postpartum period, including HIV-centric obstetric care and group prenatal care, this area represents a clear gap in the literature [40, 41]. Indeed, a recent opinion piece calls out the postpartum period as one of three important HIV care transition periods in need of further attention and study [42].

This gap in postpartum HIV care has added importance within the context of the updated Department of Health and Services (DHHS) guidelines for perinatally HIV exposed infants, which for the first time recommend collaborative decision-making and support for WWH who wish to breastfeed their infants [43]. The current guidelines contain risk mitigation recommendations, including maintenance of a suppressed viral load in the mother, provision of antiretroviral prophylaxis to the infant, and prompt identification and management of periods of increased risk such as infant thrush, maternal HIV viral blips, mastitis, and bleeding or cracked nipples [43]. Given the importance of maintaining viral suppression during breastfeeding and promptly identifying periods of increased risk, the overall rates of retention in care and viral suppression in this cohort are particularly concerning. However, it is possible that WWH who choose to breastfeed may be more likely to adhere to recommendations concerning ART use and viral load testing. Prior studies have shown that rates of ART adherence are higher during pregnancy than other periods, and there is evidence that this is at least partially due to a sense of maternal love and desire to protect the infant from acquiring HIV [44–47]. It is possible, therefore, that retention in care and viral suppression would be higher in breastfeeding mothers than in non-breastfeeding mothers, although additional studies are needed to address this specific question.

It is well-documented that engagement in the HIV care continuum varies by sociodemographic factors, including age, race and ethnicity, socioeconomic status, and HIV acquisition risk factor [48–50]. While this study illustrates the overall effectiveness of Philadelphia’s PCM program, it is possible that certain populations may receive more benefit from supportive case management services than others. Future studies, particularly qualitative work which solicits thoughts from women with lived experiences, should examine how to best identify and subsequently support women who are most at risk of falling out of care during the postpartum period. Additional work to identify the most impactful components of the PCM program is also needed, as this would facilitate the adaptation of this program to the specific settings and needs of other health jurisdictions.

In this analysis, we had access to the timing, number, and type of PCM services used by each woman in the cohort. However, quality case management should be customized to the unique needs of the client, making a reliance solely on number and/or type of encounters an insufficient way to understand the success of PCM in meeting the needs of clients. In addition, standardized definitions of service types were not available and the two PCM program providers sometimes documented services differently, making more nuanced comparisons subject to misclassification errors. To facilitate evaluation of case management programs and their impact on clinical outcomes, program administrators should consider adopting standard definitions and developing a service utilization and needs fulfillment tool to systematically track what services clients are using and the extent to which their needs are met. Such a tool would not only allow for stronger epidemiological studies, but also be a means of improving service delivery.

### Strengths and Limitations

A major strength of this study is its use of comprehensive public health data from a large US city conducting perinatal HIV exposure surveillance, greatly reducing the potential for selection bias. This study has one of the largest postpartum cohorts described in the literature and includes 12 years of birth data. Definitions and assumptions were tested through extensive sensitivity analyses, which strengthens confidence in the findings.

Despite the strengths of the study, it is subject to limitations. First, participation in the PCM program is opt-in, and we would therefore expect there to be differences between women who choose to use these services and women who do not. We controlled for this to the extent possible by considering all measured potential confounders when generating adjusted models, but there may be remaining unmeasured factors that bias the results. In particular, data on substance use, which is likely to impact both participation in PCM and engagement in the HIV care continuum, could not be included in adjusted models due to significant missingness in the data. There is also potential exposure misclassification, as we were unable to account for women who may have received non-perinatal medical case management services through individual clinics or insurance. However, this is likely to bias towards the null, meaning the true effect size may be higher than what is reported here. While the use of public health data is a strength of this study, it also represents a limitation, as the data was not originally collected for research purposes and there was substantial missingness in some variables. Finally, the PCM program may be implemented differently by site, with some having case managers incorporated into the medical care team and others utilizing community-based organization referrals. It is possible that slight differences in program administration may impact its effectiveness, and future studies should examine whether specific program configurations have different effectiveness across settings and sub-populations. Overall, we believe these limitations are offset by the robustness of the data that was available, the large sample size, and stringent methodology.

## Conclusion

WWH are uniquely vulnerable to falling out of the HIV care continuum during the postpartum period, when pre-existing barriers to care are compounded by the challenges of motherhood. Few interventions aimed at improving the health and wellbeing of WWH during this time have been reported in the literature, indicating a clear gap in patient-centered care for postpartum women and a need for ongoing research into effective interventions. Philadelphia’s PCM program significantly improves retention in care and viral suppression during the postpartum period and illustrates a commitment to improving public health through the provision of meaningful supportive services. Other cities, particularly those with high HIV prevalence, should consider adopting similar models of case management.

## Supplementary Information

Below is the link to the electronic supplementary material.

Supplementary file1 (DOCX 190 kb)

Supplementary file2 (DOCX 97 kb)

Supplementary file3 (DOCX 24 kb)

Supplementary file4 (DOCX 52 kb)

## Declarations

### Ethnical Approval

This study was approved by the City of Philadelphia's Institutional Review Board under a waiver of informed consent. The study was conducted in accordance with the standards set forth in the Declaration of Helsinki and its later amendments.

## References

[1] Lampe MA, Nesheim SR, Oladapo KL, Ewing AC, Wiener J, Kourtis AP. Achieving elimination of perinatal HIV in the United States. Pediatrics. 2023;151(5):e2022059604. 10.1542/peds.2022-059604 PMC10387171 37070379

[2] Chen JS, Pence BW, Rahangdale L, et al. Postpartum HIV care continuum outcomes in the southeastern USA. AIDS. 2019;33(4):637–44. 10.1097/QAD.0000000000002094 PMC6503530 30531320

[3] Adams JW, Brady KA, Michael YL, Yehia BR, Momplaisir FM. Postpartum engagement in HIV care: an important predictor of long-term retention in care and viral suppression. Clin Infect Dis. 2015;61(12):1880–7. 10.1093/cid/civ678 26265499

[4] Meade CM, Badell M, Hackett S, et al. HIV care continuum among postpartum women living with HIV in Atlanta. Infect Dis Obstet Gynecol. 2019;2019:8161495. 10.1155/2019/8161495 PMC6393891 30894788

[5] Patel K, Karalius B, Powis K, et al. Trends in post-partum viral load among women living with perinatal HIV infection in the USA: a prospective cohort study. Lancet HIV. 2020;7(3):e184–92. 10.1016/S2352-3018(19)30339-X PMC6956402 31870676

[6] Craw JA, Beer L, Tie Y, Jaenicke T, Shouse RL, Prejean J. Viral rebound among persons with diagnosed HIV who achieved viral suppression, United States. J Acquir Immune Defic Syndr. 2020;84(2):133–40. 10.1097/QAI.0000000000002321 PMC7604780 32084054

[7] McNairy ML, Teasdale CA, El-Sadr WM, Mave V, Abrams EJ. Mother and child both matter: reconceptualizing the prevention of mother-to-child transmission care continuum. Curr Opin HIV AIDS. 2015;10(6):403–10. 10.1097/COH.0000000000000199 PMC4707659 26352391

[8] Geter A, Sutton MY, Hubbard MD. Social and structural determinants of HIV treatment and care among black women living with HIV infection: a systematic review: 2005–2016. AIDS Care. 2018;30(4):409–16. 10.1080/09540121.2018.1426827 PMC6459180 29376409

[9] Small LA, Godoy SM, Lau C. Perceptions of healthcare accessibility and medical mistrust among Black women living with HIV in the USA. Cult Health Sex. 2022;25:1–15. 10.1080/13691058.2022.2155706 PMC10558086 36571392

[10] Buchberg MK, Fletcher FE, Vidrine DJ, et al. A mixed-methods approach to understanding barriers to postpartum retention in care among low-income. HIV-Infected Women AIDS Patient Care STDs. 2015;29(3):126–32. 10.1089/apc.2014.0227 PMC4442589 25612217

[11] Kapetanovic S, Christensen S, Karim R, et al. Correlates of perinatal depression in HIV-infected women. AIDS Patient Care STDs. 2009;23(2):101–8. 10.1089/apc.2008.0125 PMC2856494 19196032

[12] Wilson MG, Husbands W, Makoroka L, et al. Counselling, case management and health promotion for people living with HIV/AIDS: an overview of systematic reviews. AIDS Behav. 2013;17(5):1612–25. 10.1007/s10461-012-0283-1 PMC3663251 22961581

[13] Handford CD, Tynan AM, Rackal JM, Glazier RH. Setting and organization of care for persons living with HIV/AIDS. Cochrane Database Syst Rev. 2006. 10.1002/14651858.CD004348.pub2. PMC8406550 16856042

[14] Tso LS, Best J, Beanland R, et al. Facilitators and barriers in HIV linkage to care interventions: a qualitative evidence review. AIDS. 2016;30(10):1639–53. 10.1097/QAD.0000000000001101 PMC4889545 27058350

[15] Anderson EA, Momplaisir FM, Corson C, Brady KA. Assessing the impact of perinatal HIV case management on outcomes along the HIV care continuum for pregnant and postpartum women living with HIV, Philadelphia 2005–2013. AIDS Behav. 2017;21(9):2670–81. 10.1007/s10461-017-1714-9 28176167

[16] Philadelphia Department of Public Health Division of HIV Health. Surveillance Report, 2023. Philadelphia, PA December 2024.

[17] Philadelphia Department of Public Health. AIDS Activities Coordinating Office Surveillance Report, 2015. City of Philadelphia, Philadelphia 2016.

[18] Gibert CL. Treatment guidelines for the use of antiretroviral agents in HIV-infected adults and adolescents: an update. Fed Pract. 2016;33(Suppl 3):31s–6s. PMC6375413 30766213

[19] National Institute of Allergy and Infectious Diseases. Antiretroviral drug discovery and development. 2024. https://www.niaid.nih.gov/diseases-conditions/antiretroviral-drug-development. Accessed 7/18/2025.

[20] Centers for Disease Control and Prevention. Undetectable = Untransmittable. 2024. https://www.cdc.gov/global-hiv-tb/php/our-approach/undetectable-untransmittable.html. Accessed 16 Jan 2025.

[21] Cohen SM, Gray KM, Ocfemia MC, Johnson AS, Hall HI. The status of the National HIV Surveillance System, United States, 2013. Public Health Rep. 2014;129(4):335–41. 10.1177/003335491412900408 PMC4037459 24982536

[22] Dean BB, Hart RL, Buchacz K, Bozzette SA, Wood K, Brooks JT. HIV laboratory monitoring reliably identifies persons engaged in care. J Acquir Immune Defic Syndr. 2015;68(2):133–9. 10.1097/QAI.0000000000000406 PMC4657742 25383710

[23] HIV.gov. HIV care continuum. https://www.hiv.gov/federal-response/other-topics/hiv-aids-care-continuum. Accessed 21 May 2025.

[24] Centers for Disease Control and Prevention. HIV surveillance supplemental report: monitoring selected national HIV prevention and care objectives by using HIV surveillance data United States and 6 territories and freely associated states, 2022. 21 2024.

[25] Momplaisir FM, Storm DS, Nkwihoreze H, Jayeola O, Jemmott JB. Improving postpartum retention in care for women living with HIV in the United States. AIDS. 2018;32(2):133–42. 10.1097/QAD.0000000000001707 PMC5757672 29194122

[26] Kotelchuck M. An evaluation of the Kessner Adequacy of Prenatal Care Index and a proposed Adequacy of Prenatal Care Utilization Index. Am J Public Health. 1994;84(9):1414–20. 10.2105/ajph.84.9.1414 PMC1615177 8092364

[27] Higginbotham JK, Segovia LM, Rohm KL, Anderson CM, Breitenstein SM. Social Vulnerability Index and health outcomes in the United States: a systematic review. Fam Med Community Health. 2025. 10.1097/FCH.0000000000000421. PMC11832337 39807786

[28] Meyer D, Slone SE, Ogungbe O, Duroseau B, Farley JE. Impact of the COVID-19 pandemic on HIV healthcare service engagement, treatment adherence, and viral suppression in the United States: a systematic literature review. AIDS Behav. 2023;27(1):344–57. 10.1007/s10461-022-03771-w PMC9344234 35916951

[29] Zeileis A, Köll S, Graham N. Various versatile variances: an object-oriented implementation of clustered covariances in R. J Stat Softw. 2020;95(1):1–36.

[30] Venables WN, Ripley BD. Modern applied statistics with S. 4th ed. New York: Springer; 2002.

[31] Latham AH, Lugo Morales A, Barba Gutierrez E, et al. Toward improving retention in HIV care after pregnancy: lessons from a post-pandemic cohort in the United States. Front Med. 2025;12:1561490. 10.3389/fmed.2025.1561490 PMC12245896 40655100

[32] Paioni P, Aebi-Popp K, de Martinez Tejada B, et al. Viral suppression and retention in HIV care during the postpartum period among women living with HIV: a longitudinal multicenter cohort study. Lancet Reg Health. 2023;31:100656. 10.1016/j.lanepe.2023.100656 PMC10250913 37303945

[33] Dang T, Anderson J, Powell AM. Factors associated with retention in HIV care within 12 months postpartum: a retrospective cohort study. J Acquir Immune Defic Syndr. 2025;98(5):478–83. 10.1097/QAI.0000000000003593 PMC12301958 39745687

[34] Richterman A, Aitcheson N, Durnwald C, et al. Mental bandwidth is associated with HIV and viral suppression among low-income women in Philadelphia. AIDS Behav. 2025;29(2):556–61. 10.1007/s10461-024-04539-0 PMC11813681 39485620

[35] Joo JY, Huber DL. Barriers in case managers’ roles: a qualitative systematic review. West J Nurs Res. 2018;40(10):1522–42. 10.1177/0193945917728689 28862090

[36] Sebesta DS, Marx R, Liu Y. HIV prevention case management in San Francisco: barriers to successful implementation. AIDS Care. 2006;18(4):345–55. 10.1080/09540120500200641 16809112

[37] Centers for Disease Control and Prevention. Monitoring selected national HIV prevention and care objectives by using HIV surveillance data—United States and 6 territories and freely associated states, 2022. May 2024; https://www.cdc.gov/hivdata/nhss/national-hiv-prevention-and-care-outcomes.html

[38] Adhikari EH, Yule CS, Roberts SW, et al. Factors associated with postpartum loss to follow-up and detectable viremia after delivery among pregnant women living with HIV. AIDS Patient Care STDS. 2019;33(1):14–20. 10.1089/apc.2018.0117 30601060

[39] Meade CM, Badell M, Hackett S, et al. HIV care continuum among postpartum women living with HIV in Atlanta. Infect Dis Obstet Gynecol. 2019;2019(1):8161495. 10.1155/2019/8161495 PMC6393891 30894788

[40] McKinney J, Jackson J, Sangi-Haghpeykar H, et al. HIV-adapted group prenatal care: assessing viral suppression and postpartum retention in care. AIDS Patient Care STDS. 2021;35(2):39–46. 10.1089/apc.2020.0249 33571047

[41] Powell AM, DeVita JM, Ogburu-Ogbonnaya A, Peterson A, Lazenby GB. The effect of HIV-centered obstetric care on perinatal outcomes among a cohort of women living with HIV. J Acquir Immune Defic Syndr. 2017;75(4):431–8. 10.1097/QAI.0000000000001432 28452911

[42] Murray M, Khaleq F, O’Donnell EP. Considerations for select transitions of care for people with HIV: adolescent to adult, prison to society and the postpartum period. Drugs Context. 2022. 10.7573/dic.2021-8-11. PMC8903878 35310297

[43] Panel on Treatment of HIV During Pregnancy and Prevention of Perinatal Transmission. Recommendations for the use of antiretroviral drugs during pregnancy and interventions to reduce perinatal HIV transmission in the United States. Department of Health and Human Services. 2023

[44] Gertsch A, Michel O, Locatelli I, et al. Adherence to antiretroviral treatment decreases during postpartum compared to pregnancy: a longitudinal electronic monitoring study. AIDS Patient Care STDs. 2013;27(4):208–10. 10.1089/apc.2013.0005 PMC3624692 23506310

[45] Kanguya T, Koyuncu A, Sharma A, et al. Identifying barriers to ART initiation and adherence: an exploratory qualitative study on PMTCT in Zambia. PLoS ONE. a2022;17(1):e0262392. 10.1371/journal.pone.0262392 PMC8757984 35025923

[46] DiClemente-Bosco K, Weber AZ, Harrison A, et al. Empowerment in pregnancy: ART adherence among women living with HIV in Cape Town, South Africa. Soc Sci Med. 2022;296:114738. 10.1016/j.socscimed.2022.114738 PMC8882130 35078104

[47] Bardeguez AD, Lindsey JC, Shannon M, et al. Adherence to antiretrovirals among US women during and after pregnancy. J Acquir Immune Defic Syndr. 2008;48(4):408–17. 10.1097/QAI.0b013e31817bbe80 PMC2764488 18614923

[48] Desir FA, Lesko CR, Moore RD, et al. One size fits (n)one: the influence of sex, age, and sexual human immunodeficiency virus (HIV) acquisition risk on racial/ethnic disparities in the HIV care continuum in the United States. Clin Infect Dis. 2019;68(5):795–802. 10.1093/cid/ciy556 PMC6376102 30169624

[49] Kerr J, Smith A, Nzama N, et al. Systematic review of neighborhood factors impacting HIV care continuum participation in the United States. J Urban Health. 2024;101(1):31–63. 10.1007/s11524-023-00801-3 PMC10897076 38093034

[50] Dailey A, Johnson AS, Hu X, Gant Z, Lyons SJ, Adih W. Trends in HIV care outcomes among adults and adolescents—33 jurisdictions, United States, 2014–2018. J Acquir Immune Defic Syndr. 2021;88(4):333–9. 10.1097/QAI.0000000000002778 PMC8621807 34369909

