# Supplementary material for: Women in the HIV Care Continuum: Assessing Re-linking Women to Care and Maintaining Viral Suppression Postpartum in Philadelphia from 2012 to 2023
Source: AIDS Behav. 2026 Mar 12;30(10):3028–40. doi: 10.1007/s10461-026-05096-4 (PMC13669074; doi:10.1007/s10461-026-05096-4)
Supplement: Supplementary file 1 [file 10461_2026_5096_MOESM1_ESM.docx]

Consort diagram describing the individuals excluded from the study cohort. Panel A shows the birthing persons, and panel B shows the infants. The initial cohort included all birthing persons (or infants) with HIV who gave birth between January 1, 2012-December 32, 2021 and who lived, gave birth, or received perinatal and pediatric care services in Philadelphia, PA.

**A**

**B**

Women in the HIV Care Continuum: Assessing Re-Linking Women to Care and Maintaining Viral Suppression Postpartum in Philadelphia from 2012-2023

KJ Nightingale*, T Nassau, A Karaj, JH Holmes, Y Li, FM Momplaisir, KA Brady, ED Lowenthal

*Corresponding Author. [Kira.Nightingale@pennmedicine.upenn.edu](mailto:Kira.Nightingale@pennmedicine.upenn.edu)
