# Supplementary material for: Women in the HIV Care Continuum: Assessing Re-linking Women to Care and Maintaining Viral Suppression Postpartum in Philadelphia from 2012 to 2023
Source: AIDS Behav. 2026 Mar 12;30(10):3028–40. doi: 10.1007/s10461-026-05096-4 (PMC13669074; doi:10.1007/s10461-026-05096-4)
Supplement: Supplementary file 2 [file 10461_2026_5096_MOESM2_ESM.docx]

**Sensitivity analysis of the definition of PCM use**. The primary analysis defined PCM use as 2+ encounters during pregnancy through 90 days postpartum (for retention in care at 90 days), and 2+ encounters during the 90-365 days postpartum (for retention and viral suppression at 1 year). The number of encounters required to meet the definition of PCM use was varied from 1-5, and the median number of encounters (19 for retention at 90 days, 8 for retention and viral suppression at 1 year).

**Number of women meeting the criteria for PCM use as the encounter threshold is varied – 90 Day Outcome**

| **Primary Analysis**  **(2+ Encounters)** | |  | **1+ Encounter** | |  | **3+ Encounters** | |  | **4+ Encounters** | |  | **5+ Encounters** | |  | **19+ Encounters (Median)** | |
| --- | --- | --- | --- | --- | --- | --- | --- | --- | --- | --- | --- | --- | --- | --- | --- | --- |
| PCM Use | 543 |  | PCM Use | 641 |  | PCM Use | 479 |  | PCM Use | 470 |  | PCM Use | 463 |  | PCM Use | 359 |
| No PCM Use | 394 |  | No PCM Use | 296 |  | No PCM Use | 458 |  | No PCM Use | 467 |  | No PCM Use | 474 |  | No PCM Use | 578 |

**Number of women meeting the criteria for PCM use as the encounter threshold is varied – 1 Year Outcomes**

| **Primary Analysis**  **(2+ Encounters)** | |  | **1+ Encounter** | |  | **3+ Encounters** | |  | **4+ Encounters** | |  | **5+ Encounters** | |  | **8+ Encounters (Median)** | |
| --- | --- | --- | --- | --- | --- | --- | --- | --- | --- | --- | --- | --- | --- | --- | --- | --- |
| PCM Use | 407 |  | PCM Use | 490 |  | PCM Use | 392 |  | PCM Use | 379 |  | PCM Use | 368 |  | PCM Use | 246 |
| No PCM Use | 515 |  | No PCM Use | 432 |  | No PCM Use | 530 |  | No PCM Use | 543 |  | No PCM Use | 554 |  | No PCM Use | 676 |

**Retention at 90 Days Postpartum**

| **Primary Analysis - 2 Encounters** | | | |  | **1+ Encounters** | | | |  | **3+ Encounters** | | | |
| --- | --- | --- | --- | --- | --- | --- | --- | --- | --- | --- | --- | --- | --- |
|  | **aOR** | **95% CI** | **P-value** |  |  | **aOR** | **95% CI** | **P-value** |  |  | **aOR** | **95% CI** | **P-value** |
| **PCM Use** |  |  |  |  | **PCM Use** |  |  |  |  | **PCM Use** |  |  |  |
| No | Ref |  |  |  | No | Ref |  |  |  | No | Ref |  |  |
| Yes | 1.403 | 1.030-1.913 | **0.03** |  | Yes | 1.637 | 1.172-2.288 | **<0.01** |  | Yes | 1.307 | 0.952-1.795 | 0.10 |

| **4+ Encounters** | | | |  | **5+ Encounters** | | | |  | **12+ Encounters (Median)** | | | |
| --- | --- | --- | --- | --- | --- | --- | --- | --- | --- | --- | --- | --- | --- |
|  | **aOR** | **95% CI** | **P-value** |  |  | **aOR** | **95% CI** | **P-value** |  |  | **aOR** | **95% CI** | **P-value** |
| **PCM Use** |  |  |  |  | **PCM Use** |  |  |  |  | **PCM Use** |  |  |  |
| No | Ref |  |  |  | No | Ref |  |  |  | No | Ref |  |  |
| Yes | 1.317 | 0.958-1.809 | 0.09 |  | Yes | 1.320 | 0.962-1.813 | 0.09 |  | Yes | 1.421 | 1.032-1.958 | **0.03** |

**Retention at 1 Year Postpartum**

| **Primary Analysis - 2 Encounters** | | | |  | **1+ Encounters** | | | |  | **3+ Encounters** | | | |
| --- | --- | --- | --- | --- | --- | --- | --- | --- | --- | --- | --- | --- | --- |
|  | **aOR** | **95% CI** | **P-value** |  |  | **aOR** | **95% CI** | **P-value** |  |  | **aOR** | **95% CI** | **P-value** |
| **PCM Use** |  |  |  |  | **PCM Use** |  |  |  |  | **PCM Use** |  |  |  |
| No | Ref |  |  |  | No | Ref |  |  |  | No | Ref |  |  |
| Yes | 1.576 | 1.163-2.136 | **<0.01** |  | Yes | 1.806 | 1.335-2.444 | **<0.01** |  | Yes | 1.669 | 1.229-2.267 | **<0.01** |

| **4+ Encounters** | | | |  | **5+ Encounters** | | | |  | **20+ Encounters (Median)** | | | |
| --- | --- | --- | --- | --- | --- | --- | --- | --- | --- | --- | --- | --- | --- |
|  | **aOR** | **95% CI** | **P-value** |  |  | **aOR** | **95% CI** | **P-value** |  |  | **aOR** | **95% CI** | **P-value** |
| **PCM Use** |  |  |  |  | **PCM Use** |  |  |  |  | **PCM Use** |  |  |  |
| No | Ref |  |  |  | No | Ref |  |  |  | No | Ref |  |  |
| Yes | 1.743 | 1.287-2.361 | **<0.01** |  | Yes | 1.859 | 1.360-2.541 | **<0.01** |  | Yes | 2.230 | 1.558-3.193 | **<0.01** |

**Viral Suppression at 1 Year Postpartum**

| **Primary Analysis - 2 Encounters** | | | |  | **1+ Encounters** | | | |  | **3+ Encounters** | | | |
| --- | --- | --- | --- | --- | --- | --- | --- | --- | --- | --- | --- | --- | --- |
|  | **aOR** | **95% CI** | **P-value** |  |  | **aOR** | **95% CI** | **P-value** |  |  | **aOR** | **95% CI** | **P-value** |
| **PCM Use** |  |  |  |  | **PCM Use** |  |  |  |  | **PCM Use** |  |  |  |
| No | Ref |  |  |  | No | Ref |  |  |  | No | Ref |  |  |
| Yes | 1.412 | 1.060-1.881 | **0.02** |  | Yes | 1.491 | 1.109-2.005 | **<0.01** |  | Yes | 1.492 | 1.119-1.989 | **<0.01** |

| **4+ Encounters** | | | |  | **5+ Encounters** | | | |  | **20+ Encounters (Median)** | | | |
| --- | --- | --- | --- | --- | --- | --- | --- | --- | --- | --- | --- | --- | --- |
|  | **aOR** | **95% CI** | **P-value** |  |  | **aOR** | **95% CI** | **P-value** |  |  | **aOR** | **95% CI** | **P-value** |
| **PCM Use** |  |  |  |  | **PCM Use** |  |  |  |  | **PCM Use** |  |  |  |
| No | Ref |  |  |  | No | Ref |  |  |  | No | Ref |  |  |
| Yes | 1.562 | 1.171-2.082 | **<0.01** |  | Yes | 1.582 | 1.184-2.114 | **<0.01** |  | Yes | 1.482 | 1.075-2.041 | **0.02** |

Women in the HIV Care Continuum: Assessing Re-Linking Women to Care and Maintaining Viral Suppression Postpartum in Philadelphia from 2012-2023

KJ Nightingale*, T Nassau, A Karaj, JH Holmes, Y Li, FM Momplaisir, KA Brady, ED Lowenthal

*Corresponding Author. [Kira.Nightingale@pennmedicine.upenn.edu](mailto:Kira.Nightingale@pennmedicine.upenn.edu)
