# Supplementary material for: Women in the HIV Care Continuum: Assessing Re-linking Women to Care and Maintaining Viral Suppression Postpartum in Philadelphia from 2012 to 2023
Source: AIDS Behav. 2026 Mar 12;30(10):3028–40. doi: 10.1007/s10461-026-05096-4 (PMC13669074; doi:10.1007/s10461-026-05096-4)
Supplement: Supplementary file 3 [file 10461_2026_5096_MOESM3_ESM.docx]

**Sensitivity analysis of the timing of PCM use.** Adjusted analyses assessing the impact of PCM use on retention in care and viral suppression at 1 year postpartum, defining PCM use as 2+ encounters from 270-365 days postpartum. The cohort includes women living with HIV who lived, gave birth, or received prenatal/pediatric care in Philadelphia, PA between January 1, 2012-December 31, 2023

|  | **Retention in Care 1 Year Postpartum** | | | **Viral Suppression at 1 Year Postpartum** | | | **Viral Suppression at 1 Year (Carry Forward Last Lab)** | | |
| --- | --- | --- | --- | --- | --- | --- | --- | --- | --- |
|  | **aOR** | **95% CI** | **P-value** | **aOR** | **95% CI** | **P-value** | **aOR** | **95% CI** | **P-value** |
| **PCM Use** |  |  |  |  |  |  |  |  |  |
| No | Ref |  |  | Ref |  |  | Ref |  |  |
| Yes | 2.454 | 1.751-3.440 | **<0.01** | 1.855 | 1.361-2.530 | **<0.01** | 2.315 | 1.631-3.285 | **<0.01** |
| **Race** |  |  |  |  |  |  |  |  |  |
| White | Ref |  |  | Ref |  |  | Ref |  |  |
| Black/African American | 1.297 | 0.765-2.197 | 0.33 | 0.829 | 0.474-1.451 | 0.51 | 0.665 | 0.389-1.136 | 0.14 |
| Hispanic/Latino | 1.296 | 0.648-2.590 | 0.46 | 0.822 | 0.406-1.662 | 0.58 | 0.509 | 0.260-0.998 | **0.05** |
| Other | 1.064 | 0.485-2.335 | 0.88 | 0.985 | 0.452-2.143 | 0.97 | 0.800 | 0.338-1.893 | 0.61 |
| **Mother's Birth Country** |  |  |  |  |  |  |  |  |  |
| US Born | Ref |  |  | Ref |  |  | Ref |  |  |
| Non-US Born | 1.645 | 1.061-2.551 | **0.03** | 0.981 | 0.671-1.435 | 0.92 | 1.219 | 0.787-1.890 | 0.38 |
| Unknown | 1.215 | 0.704-2.099 | 0.48 | 1.223 | 0.754-1.983 | 0.42 | 1.025 | 0.615-1.710 | 0.92 |
| **Philadelphia Resident** |  |  |  |  |  |  |  |  |  |
| No | Ref |  |  | Ref |  |  | Ref |  |  |
| Yes | 2.150 | 1.436-3.218 | **<0.01** | 1.844 | 1.227-2.770 | **<0.01** | 1.297 | 0.860-1.957 | 0.22 |
| **Social Vulnerability Index** | 0.767 | 0.368-1.601 | 0.48 | 0.790 | 0.392-1.595 | 0.51 | 1.053 | 0.515-2.152 | 0.89 |
| **Prior Pregnancy w/ HIV** |  |  |  |  |  |  |  |  |  |
| No | Ref |  |  | Ref |  |  | Ref |  |  |
| Yes | 0.908 | 0.664-1.240 | 0.54 | 1.240 | 0.921-1.670 | 0.16 | 1.419 | 1.021-1.971 | **0.04** |
| **Maternal HIV Risk** |  |  |  |  |  |  |  |  |  |
| Heterosexual Contact | Ref |  |  | Ref |  |  | Ref |  |  |
| IV Drug Use with/without Heterosexual Contact | 1.110 | 0.675-1.826 | 0.68 | 0.337 | 0.185-0.614 | **<0.01** | 0.555 | 0.334-0.922 | **0.02** |
| Perinatal exposure | 2.966 | 1.453-6.056 | **<0.01** | 1.035 | 0.549-1.953 | 0.91 | 0.937 | 0.522-1.681 | 0.83 |
| Other | 1.310 | 0.528-3.246 | 0.56 | 0.725 | 0.215-2.449 | 0.60 | 0.441 | 0.149-1.305 | 0.14 |
| **Detectable VL at Delivery** |  |  |  |  |  |  |  |  |  |
| No | Ref |  |  | Ref |  |  | Ref |  |  |
| Yes | 0.569 | 0.381-0.849 | **<0.01** | 0.173 | 0.099-0.304 | **<0.01** | 0.120 | 0.074-0.193 | **<0.01** |

Women in the HIV Care Continuum: Assessing Re-Linking Women to Care and Maintaining Viral Suppression Postpartum in Philadelphia from 2012-2023

KJ Nightingale*, T Nassau, A Karaj, JH Holmes, Y Li, FM Momplaisir, KA Brady, ED Lowenthal

*Corresponding Author. [Kira.Nightingale@pennmedicine.upenn.edu](mailto:Kira.Nightingale@pennmedicine.upenn.edu)
