# Supplementary material for: Women in the HIV Care Continuum: Assessing Re-linking Women to Care and Maintaining Viral Suppression Postpartum in Philadelphia from 2012 to 2023
Source: AIDS Behav. 2026 Mar 12;30(10):3028–40. doi: 10.1007/s10461-026-05096-4 (PMC13669074; doi:10.1007/s10461-026-05096-4)
Supplement: Supplementary file 4 [file 10461_2026_5096_MOESM4_ESM.docx]

**Adjusted Analyses Including a COVID-19 Indicator Variable**

The COVID period was defined as occurring between 3/16/2020, when the statewide shutdown of non-essential businesses went into effect in Pennsylvania, and 5/11/2023, when the federal public health emergency ended.^1, 2^ Outcomes were classified as having occurred during the COVID period when the 90-day or 1-year postpartum dates fell between 3/16/2020 and 5/11/2023.

There were no significant differences in PCM use based on whether or not the infant’s date of birth fell during the COVID-19 period (p=0.45 for PCM use during pregnancy through 90 days postpartum, and p=0.86 for PCM use from 90 days through 1 year postpartum). There was also no significant difference in PCM use based on whether the 90-day or 1-year outcome date occurred during the COVID-19 period (p=0.25 and p=0.26, respectively).

Adjusted analyses assess the impact of PCM use on the indicated outcome in a cohort of women living with HIV who lived, gave birth, or received prenatal/pediatric care in Philadelphia, PA between January 1, 2012-December 31, 2023.

**Engagement in Care at 90 Days Postpartum**

|  | **aOR** | **95% CI** | **P-value** |
| --- | --- | --- | --- |
| **PCM Use** |  |  |  |
| No | Ref |  |  |
| Yes | 1.440 | 1.093-1.900 | **0.01** |
| **90 Day Postpartum During COVID-19** |  |  |  |
| No | Ref |  |  |
| Yes | 0.719 | 0.521-0.989 | **0.04** |
| **Social Vulnerability Index** | 0.844 | 0.430-1.650 | 0.62 |
| **Race** |  |  |  |
| White | Ref |  |  |
| Black/African American | 1.548 | 0.947-2.554 | 0.08 |
| Hispanic/Latino | 1.316 | 0.703-2.476 | 0.39 |
| Other | 2.577 | 1.219-5.559 | **0.01** |
| **Mother's Birth Country** |  |  |  |
| US Born | Ref |  |  |
| Non-US Born | 1.487 | 1.038-2.140 | **0.03** |
| Unknown | 0.831 | 0.531-1.291 | 0.41 |
| **Philadelphia Resident** |  |  |  |
| No | Ref |  |  |
| Yes | 1.788 | 1.250-2.569 | **<0.01** |
| **Maternal HIV Risk** |  |  |  |
| Heterosexual Contact | Ref |  |  |
| IV Drug Use with/without Heterosexual Contact | 1.346 | 0.846-2.152 | 0.21 |
| Perinatal Exposure |  |  |  |
| Other | 2.915 | 1.797-4.849 | **<0.01** |

|  | **Retention in Care at 1 Year Postpartum** | | | **Viral Suppression at 1 Year Postpartum** | | |
| --- | --- | --- | --- | --- | --- | --- |
|  | **aOR** | **95% CI** | **P-value** | **aOR** | **95% CI** | **P-value** |
| **PCM Use** |  |  |  |  |  |  |
| No | Ref |  |  | Ref |  |  |
| Yes | 1.673 | 1.238-2.266 | **<0.01** | 1.465 | 1.097-1.959 | **0.01** |
| **1 Year Postpartum During COVID-19** |  |  |  |  |  |  |
| No | Ref |  |  | Ref |  |  |
| Yes | 0.486 | 0.338-0.697 | **<0.01** | 0.802 | 0.559-1.146 | 0.23 |
| **Race** |  |  |  |  |  |  |
| White | Ref |  |  | Ref |  |  |
| Black/African American | 1.272 | 0.759-2.123 | 0.36 | 0.853 | 0.500-1.459 | 0.56 |
| Hispanic/Latino | 1.270 | 0.653-2.490 | 0.48 | 0.845 | 0.428-1.661 | 0.62 |
| Other | 0.834 | 0.389-1.798 | 0.64 | 0.869 | 0.391-1.913 | 0.73 |
| **Mother's Birth Country** |  |  |  |  |  |  |
| US Born | Ref |  |  | Ref |  |  |
| Non-US Born | 1.445 | 0.980-2.154 | 0.07 | 0.903 | 0.626-1.300 | 0.58 |
| Unknown | 1.073 | 0.665-1.756 | 0.77 | 1.167 | 0.721-1.887 | 0.53 |
| **Prior Pregnancy w/ HIV** |  |  |  |  |  |  |
| No | Ref |  |  | Ref |  |  |
| Yes | 1.277 | 0.915-1.792 | 0.15 | 1.837 | 1.223-2.761 | **<0.01** |
| **Philadelphia Resident** |  |  |  |  |  |  |
| No | Ref |  |  | Ref |  |  |
| Yes | 2.228 | 1.536-3.243 | **<0.01** | 1.397 | 1.013-1.930 | **0.042** |
| **Social Vulnerability Index** | 1.483 | 0.771-2.836 | 0.24 | 1.306 | 0.686-2.506 | 0.42 |
| **Maternal HIV Risk** |  |  |  |  |  |  |
| Heterosexual Contact | Ref |  |  | Ref |  |  |
| IV Drug Use with/without Heterosexual Contact | 1.112 | 0.682-1.835 | 0.8 | 0.341 | 0.193-0.585 | **<0.01** |
| Perinatal Exposure | 2.812 | 1.541-5.468 | **<0.01** | 0.986 | 0.584-1.660 | 0.96 |
| Other | 1.557 | 0.465-6.227 | 0.49 | 0.777 | 0.222-2.527 | 0.68 |
| **Detectable VL at Delivery** |  |  |  |  |  |  |
| No | Ref |  |  | Ref |  |  |
| Yes | 0.500 | 0.335-0.747 | **<0.01** | 0.160 | 0.089-0.271 | **<0.01** |

**References:**

1. National Governors Association. Pennsylvania – Coronavirus State Actions 2020. Access date: 7/11/2025. Available from: https://www.nga.org/coronavirus-state-actions/pennsylvania/.

2. US Department of Health and Human Services. COVID-19 Public Health Emergency 2023. Access date: 7/11/2025. Available from: https://www.hhs.gov/coronavirus/covid-19-public-health-emergency/index.html.

Women in the HIV Care Continuum: Assessing Re-Linking Women to Care and Maintaining Viral Suppression Postpartum in Philadelphia from 2012-2023

KJ Nightingale*, T Nassau, A Karaj, JH Holmes, Y Li, FM Momplaisir, KA Brady, ED Lowenthal

*Corresponding Author. [Kira.Nightingale@pennmedicine.upenn.edu](mailto:Kira.Nightingale@pennmedicine.upenn.edu)
